# Infective Endocarditis Among Women Who Inject Drugs

**DOI:** 10.1001/jamanetworkopen.2024.37861

**Published:** 2024-10-04

**Authors:** Janica A. Adams, Cara Spence, Esfandiar Shojaei, Priyadarshini Thandrasisla, Anmol Gupta, Yun-Hee Choi, Stuart Skinner, Michael Silverman

**Affiliations:** 1 Division of Infectious Diseases, Department of Medicine, College of Medicine, University of Saskatchewan, Saskatoon, Saskatchewan, Canada; 2 Wellness Wheel Medical Clinic & Indigenous Community Research Network, Regina, Saskatchewan, Canada; 3 Lawson Health Research Institute, London, Ontario, Canada; 4 Department of Epidemiology and Biostatistics, Schulich School of Medicine and Dentistry, Western University, London, Ontario, Canada; 5 College of Medicine, University of Saskatchewan, Saskatoon, Saskatchewan, Canada; 6 Division of Infectious Diseases, Department of Medicine, College of Medicine, University of Saskatchewan, Regina, Saskatchewan, Canada; 7 Department of Indigenous Health and Wellness, College of Medicine, University of Saskatchewan, Regina, Saskatchewan, Canada; 8 Division of Infectious Diseases, Department of Medicine, St Joseph’s Hospital, London, Ontario, Canada; 9 Division of Infectious Diseases, Department of Medicine, Western University, London, Ontario, Canada

## Abstract

**Importance:**

In the US and Canada, women comprise approximately one-third of people who inject drugs (PWID); however, clinical characteristics and outcomes of injection drug use complications in women are poorly described.

**Objective:**

To identify clinical characteristics and outcomes of infective endocarditis (IE) among women who inject drugs (WWID).

**Design, Setting, and Participants:**

This is a retrospective cohort study of PWID with definite IE (per 2023 Duke–International Society for Cardiovascular Infectious Diseases criteria) admitted from April 5, 2007, to March 15, 2018, at 5 tertiary-care hospitals in London, Ontario, and Regina, Saskatchewan, Canada. Data were analyzed from June 1, 2023, to August 2, 2024. Descriptive analyses were conducted for baseline characteristics at index hospitalization and stratified by sex.

**Main Outcomes and Measures:**

The primary outcome was the difference in 5-year survival between female and male PWID with IE. The secondary outcome was 1-year survival. Multivariable time-dependent Cox proportional hazards regression analyses were conducted for variables of clinical importance to evaluate 5-year mortality.

**Results:**

Of 430 PWID with IE, 220 (51.2%) were women; of 332 non-PWID with IE, 101 (30.4%) were women. WWID with IE were younger than men (median [IQR] age, 31.5 [27.0-38.5] vs 38.5 [31.0-49.0] years), and 11 of 220 (5.0%) were pregnant at index hospitalization, although only 12 of 220 (5.5%) had contraceptive use documented. Women had a larger proportion of right-sided IE than men (158 of 220 women [71.8%] vs 113 of 210 men [53.8%]). WWID living in urban areas had higher mortality than WWID in rural areas (adjusted hazard ratio [aHR], 2.70; 95% CI, 1.15-6.34; *P* = .02). Overall mortality was lower among PWID referred for substance use disorder counseling in centers with inpatient services compared with centers with only outpatient referrals (aHR, 0.29; 95% CI, 0.17-0.51; *P* < .001). Overall mortality was lower with right-sided heart disease for both women (aHR, 0.44; 95% CI, 0.27-0.71; *P* < .001) and men (aHR, 0.22; 95% CI, 0.10-0.50; *P* < .001) and was higher with congestive heart failure for both women (aHR, 2.32; 95% CI, 1.29-4.18; *P* = .005) and men (aHR, 1.73; 95% CI, 1.07-2.79; *P* = .02).

**Conclusions and Relevance:**

In this cohort of PWID with IE, women were overrepresented. Reasons for women’s disproportionately high IE incidence need further study. Inpatient substance use disorder services, contraception counseling, and enhanced social support for WWID living in urban areas need to be prioritized.

## Key Points

**Question:**

What demographic and clinical factors are associated with mortality among women who inject drugs (WWID) with infective endocarditis (IE)?

**Findings:**

In this retrospective cohort study of 430 people who inject drugs with IE, women comprised more than 50% of the group. WWID with IE who lived in urban settings had higher hazard of mortality than woman in rural settings, and inpatient substance use disorder counseling was associated with better long-term survival.

**Meaning:**

WWID have a disproportionately high incidence of IE, suggesting that social support for WWID in urban settings, inpatient substance use disorder counseling, and contraceptive services need prioritization.

## Teaser

This cohort study examines the clinical characteristics and outcomes of infective endocarditis among women who inject drugs.

## Introduction

Infective endocarditis (IE) is an infection of the endocardium and heart valves caused by microorganisms entering the bloodstream.^1^ The opioid epidemic has been associated with an increase in hospitalizations for IE.^2,3,4^ In 2020, the estimated global population of people who inject drugs (PWID) was more than 11 million.^5^ Injection drug use contributes to extensive morbidity and mortality in Canada,^6^ resulting in more than 44 000 opioid-related deaths from 2016 to 2024.^7^ The population of PWID in Canada increased nearly 33% from 2011 to 2016,^6^ with women accounting for one-third (32.7%) of total injection drug use numbers nationally,^8^ similar to US and Australasian estimates.^5,9,10^ In the US, the number of PWID increased 5-fold from 2011 to 2018.^10,11^ PWID hospitalizations for IE are increasing in rural areas, exceeding those in urban areas across the US.^12^ Data on outcomes in PWID-associated IE in rural vs urban areas are not yet available. Women comprise approximately 33% of non-PWID IE cases,^2,13^ whereas the literature suggests that 45% to 55% of IE cases among PWID are women.^2,3,4,14,15^ Despite this difference, clinical presentation and outcomes among women who inject drugs (WWID) with IE have not been previously evaluated. A shortage of health care workers trained in substance use disorder (SUD) management has led to limitations in access to inpatient SUD services.^16^ We aimed to determine whether women are disproportionately represented within a cohort of PWID with IE, describe the presentation and long-term mortality of IE among WWID, assess the association of rurality with long-term mortality in WWID with IE, and determine the association of inpatient SUD counseling with long-term mortality.

## Methods

### Study Design and Patient Population

This retrospective cohort study consists of patients aged 18 years or older, admitted from April 5, 2007, to March 15, 2018, to any of 5 hospitals in Canada, including 3 hospitals in London, Ontario, and 2 hospitals in in Regina, Saskatchewan. We reviewed the medical records of patients with a discharge diagnosis of IE. The Strengthening the Reporting of Observational Studies in Epidemiology (STROBE) reporting guideline^17^ criteria were used. Infectious disease specialists in London (E.S. and M.S.) and Regina (A.G. and S.S.) reviewed case data to ensure cases met the 2023 Duke–International Society for Cardiovascular Infectious Diseases (ISCVID) criteria for definite IE.^18^ Ethics approval was obtained from the Lawson Research Institute’s Review Board in London and the Saskatchewan Health Authority Research Ethics Board. The need for informed consent was waived because the data were observational and deidentified. Preliminary data regarding the London cohort were previously published in a study of bloodstream infections among patients undergoing therapy for IE^19^; however, follow-up was extended to 5 years and extended to include patients from Regina. The primary outcome of interest was 5-year mortality. Patient death was ascertained using medical records and obituaries.^20^ A citywide medical record in each of Regina and London allowed long-term follow-up. Study measures are described in the eMethods in Supplement 1.

### Statistical Analysis

We conducted descriptive analyses comparing baseline characteristics of male and female PWID at index hospitalization. We presented categorical variables using frequencies (percentages) and continuous variables using medians (IQRs). Missing data were recorded where applicable. Pregnancy among women and contraceptive use or nonuse were described. Pearson χ^2^ and Fisher exact tests were used to assess distribution of categorical variables where appropriate.

Multivariable time-dependent Cox proportional hazards regression analyses were conducted using data from the first IE episode for clinically important variables associated with all-cause 5-year mortality among patients with IE. Model variables were selected a priori and included age, sex, province, urbanicity, location of infection, SUD counseling, and congestive heart failure (CHF). The eFigure in Supplement 1 illustrates the causal diagram. Formal interaction tests were conducted for sex and urbanicity, sex and referral to SUD counseling, and province and referral to SUD counseling.

We excluded cardiac surgery (hereafter, surgery) in the models because of the concern for overcorrection of other variables, such as right-sided infection and CHF. Sensitivity analyses (with adjustment for surgery, and interactions between surgery and CHF, right-sided infection, and sex) were conducted to determine whether surgery acted as an effect modifier (eMethods in Supplement 1). Surgery was treated as time dependent to avoid immortal time bias.^19,21^ Time at risk was defined as time from index admission to time of death. The proportional hazards assumption was assessed using scaled Schoenfeld residuals. A step function, whereby data were categorized into 3 periods (5-year mortality, <90 days, 90-365 days, and >365 days; 1-year mortality, <90 days, 90-180 days, and >180 days), was applied to variables violating the proportional hazards assumption.^22^ Substantive model compatible fully conditional specification imputation was used to address missing data.^23,24^ Analyses were conducted using SAS OnDemand for Academics statistical software release 3.81 (Enterprise Edition; SAS Institute) and R statistical software version 4.3.2 (R Project for Statistical Computing). Statistical significance was determined using 2-sided 95% CIs and *P* < .05. Data were analyzed from June 1, 2023, to August 2, 2024.

## Results

### Baseline Statistics

Seven hundred sixty-four patients (321 women and 441 men) met the 2023 Duke-ISCVID criteria for definite IE.^18^ In total, 430 of 764 patients (56.0%) identified as PWID, including 220 women (51.2%) and 210 men (48.8%), as shown in the Figure. Women comprised a larger proportion of PWID than non-PWID (51.2% [220 of 430 individuals] vs 30.4% [101 of 332 individuals]; χ^2^_1_ = 33.1; *P* < .001) (eTable 1 and eTable 2 in Supplement 1).

**Figure.**
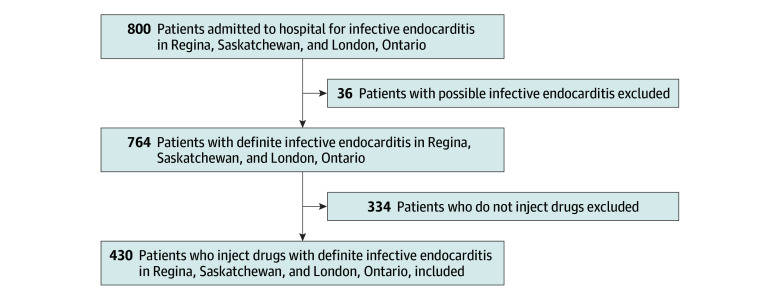
Patient Enrollment Flowchart

Table 1 shows baseline characteristics of the entire cohort, at index hospitalization. Women were younger than men (median [IQR] age, 31.5 [27.0-38.5] vs 38.5 [31.0-49.0] years). Tricuspid valve vegetation (166 women [76.2%] vs 118 men [58.1%]), right-sided infection (158 women [71.8%] vs 113 men [53.8%]), septic pulmonary emboli (142 women [69.3%] vs 105 men [57.1%]), and being referred to SUD services (102 women [47.0%] vs 66 men [31.6%]) were more commonly observed among women than men. Men were more likely than women to present with CHF (43 men [22.4%] vs 29 women [13.6%]) and myocardial abscess (16 men [8.0%] vs 7 women [3.3%]) and to have undergone surgery (41 men [19.5%] vs 25 women [11.4%]). The incidence of recurrent IE did not differ between men and women (eTable 3 in Supplement 1).

**Table 1.** Baseline Characteristics of Cohort at Index Hospitalization, by Sex^a^

Characteristic	Participants, No. (%)		
	Total (N = 430)	Women (n = 220)	Men (n = 210)
Age, median (IQR), y	35.0 (28.0-43.0)	31.5 (27.0-38.5)	38.5 (31.0-49.0)
Province of residence			
Regina	120 (27.9)	69 (31.4)	51 (24.3)
London	310 (72.1)	151 (68.6)	159 (75.7)
Indigenous status^b^			
No	47 (42.0)	20 (31.8)	27 (55.1)
Yes	65 (58.0)	43 (68.3)	22 (44.9)
Unknown^c^	318	157	161
Urbanicity			
Rural	57 (13.3)	32 (14.7)	25 (11.9)
Urban	371 (86.7)	186 (85.3)	185 (88.1)
Unknown^c^	2	2	0
Cardiac site of infection			
Tricuspid^d^	284 (67.5)	166 (76.2)	118 (58.1)
Pulmonic	6 (1.4)	5 (2.3)	1 (0.5)
Mitral	83 (19.7)	34 (15.6)	49 (24.1)
Aortic	77 (18.3)	27 (12.4)	50 (24.6)
Right ventricle or atrium	6 (1.4)	4 (1.8)	2 (1.0)
Device^d^	1 (0.2)	0	1 (0.5)
Site of infection			
Right-sided	271 (63.0)	158 (71.8)	113 (53.8)
Left-sided	118 (27.4)	40 (18.2)	78 (37.1)
Bilateral	24 (5.6)	15 (6.8)	9 (4.3)
Echo-negative	17 (4.0)	7 (3.2)	10 (4.8)
Infectious organism			
Methicillin-sensitive *Staphylococcus aureus*	222 (52.2)	118 (54.1)	104 (50.2)
Methicillin-resistant *Staphylococcus aureus*	100 (23.5)	56 (25.7)	44 (21.3)
Other^e^	103 (24.2)	44 (20.2)	59 (28.5)
Unknown^c^	5	2	3
Cardiac complications^f^			
Myocardial abscess	23 (5.5)	7 (3.3)	16 (8.0)
Aortic root abscess	14 (3.3)	5 (2.3)	9 (4.3)
Congestive heart failure^g^	72 (17.7)	29 (13.6)	43 (22.4)
Conduction delay	12 (2.8)	5 (2.3)	7 (3.3)
Vascular complications^f^			
Ischemic stroke	67 (16.8)	30 (14.2)	37 (19.6)
Intracerebral hemorrhage	32 (8.3)	15 (7.4)	17 (9.2)
Mycotic aneurysm	16 (4.1)	7 (3.5)	9 (4.9)
Septic pulmonary emboli	247 (63.5)	142 (69.3)	105 (57.1)
Admitted to intensive care unit			
No	276 (64.2)	139 (63.2)	137 (65.2)
Yes	154 (35.8)	81 (36.8)	73 (34.8)
Referred to substance use disorder counseling			
No	258 (60.6)	115 (53.0)	143 (68.4)
Yes	168 (39.4)	102 (47.0)	66 (31.6)
Unknown^c^	4	3	1
Cardiac surgery			
No	364 (84.7)	195 (88.6)	169 (80.5)
Yes	66 (15.4)	25 (11.4)	41 (19.5)

^a^ The first hospitalization is defined as that occurring during the first episode. Refer to eTable 2 in Supplement 1 for data regarding HIV status, hepatitis C status, category of injection substances used, length of hospital stay, peripherally inserted central catheter misuse, route of antibiotic administration, left against medical advice, infectious disease consultation, and causative organism.

^b^ Data for Indigenous status were available from Regina, Saskatchewan, only.

^c^ Unknown numbers were not included in calculations of percentages.

^d^ One male participant had an implanted defibrillator that also demonstrated tricuspid valve vegetation.

^e^ Refer to eTable 2 in Supplement 1 for a complete list of causative organisms.

^f^ Complications were identified within the index hospitalization.

^g^ Heart failure was diagnosed by the attending team.

### Mortality Among All PWID

One-year follow-up data were available for 415 of 430 patients (96.5%), of whom 49 of 211 women (23.2%) and 67 of 204 men (32.8%) died within 1 year of index admission (χ^2^_1_ = 4.8; *P* = .03) (eTable 4 in Supplement 1). Five-year follow-up data were available for 347 of 430 patients (80.7%). Five-year mortality was 45.7% (80 of 175 individuals) among women and 51.7% (89 of 172 individuals) among men (χ^2^_1_ = 1.3; *P* = .26), for an overall 5-year mortality rate of 48.7%.

Patients with 1-year follow-up and those lost to follow-up did not differ by age, sex, province, urbanicity, site of infection, referral to SUD counseling, CHF and surgery (eTable 5 in Supplement 1). Patients with 5-year follow-up and those lost to follow-up differed by province (χ^2^_1_ = 26.3; *P* < .001) and CHF (χ^2^_1_ = 4.08; *P* = .04) (eTable 5 in Supplement 1).

We observed an interaction between province and referral to SUD counseling (*P* for interaction = .02), suggesting among all PWID, residing in London and surrounding areas compared with Regina and surrounding areas decreased the hazard of 5-year mortality among PWID referred to SUD counseling (adjusted HR [aHR], 0.29; 95% CI, 0.17-0.51; *P* < .001) (Table 2). This survival benefit was not observed for patients not referred to SUD counseling. There was no interaction between SUD and sex. Sex had a significant interaction with urbanicity (*P* for interaction = .02) whereby residing in urban areas, compared with rural areas, increased the hazard of 5-year mortality only among women (aHR, 2.70; 95% CI, 1.15-6.34; *P* = .02). Right-sided infection, compared with left-sided, bilateral, echo-negative infection, was associated with a lower hazard of 5-year mortality only within 90 days following index hospitalization (aHR, 0.24; 95% CI, 0.13-0.41; *P* < .001). Having CHF increased the hazard of 5-year mortality (aHR, 1.79; 95% CI, 1.24-2.59; *P* = .002) among patients.

**Table 2.** Multivariable Time-Dependent Cox Proportional for Factors Associated With 5-Year Mortality in Persons Who Inject Drugs

Variable	aHR (95% CI)^a^	*P* value
Age at time of admission	1.01 (1.00-1.03)	.13
Province of residence by referral to SUD counseling		.02^b^
Residing in London, ON, and surrounding areas vs Regina, SK, and surrounding areas among those who were referred to SUD counseling	0.29 (0.17-0.51)	<.001
Residing in London, ON, and surrounding areas vs Regina, SK, and surrounding areas among those who were not referred to SUD counseling	0.69 (0.44-1.08)	.11
Sex by urbanicity		.03^b^
Women vs men in urban areas	1.14 (0.79-1.67)	.48
Women vs men in rural areas	0.37 (0.14-1.00)	.05
Residing in urban areas vs rural areas among women	2.70 (1.15-6.34)	.02
Residing in urban areas vs rural areas among men	0.88 (0.47-1.64)	.68
Right-sided infection (reference, left-sided, bilateral, echo negative) by time		.003^b^
Right-sided infection <90 d	0.24 (0.13-0.41)	<.001
Right-sided infection 90-365 d	0.86 (0.42-1.75)	.68
Right-sided infection >365 d	0.73 (0.40-1.32)	.30
Congestive heart failure (reference, no)	1.79 (1.24-2.59)	.002

Abbreviations: aHR, adjusted hazard ratio; ON, Ontario; SK, Saskatchewan; SUD, substance use disorder.

^a^ aHRs were generated from a time-dependent multivariable Cox proportional hazards regression model. The multivariable model was adjusted for age at time of admission, interaction between referral to SUD counseling and time, interaction between right-sided infection and time, congestive heart failure, interaction between urbanicity and sex, and interaction between province of residence and referral to SUD counseling.

^b^ *P* values were derived from the log-likelihood ratio suggesting significant interaction term.

### Mortality Among WWID

Residing in London, compared with Regina, decreased the hazard of 5-year mortality in women by 54% (aHR, 0.46; 95% CI, 0.28-0.77; *P* = .003) (Table 3). Right-sided infection, compared with left-sided, bilateral, echo-negative infection, decreased the hazard of 5-year mortality (aHR, 0.44; 95% CI, 0.27-0.71; *P* < .001). Women residing in urban areas had an increased hazard of 5-year mortality (aHR, 3.03; 95% CI, 1.26-7.28; *P* = .01) compared with women residing in rural areas. Women with CHF had an increased hazard of 5-year mortality (aHR, 2.32; 95% CI, 1.29-4.18; *P* = .005) compared with women without CHF.

**Table 3.** Multivariable Cox Proportional for Factors Associated With 5-Year Mortality in Women Who Inject Drugs

Variable	aHR (95% CI)^a^	*P* value
Age at time of admission	1.02 (0.99-1.05)	.12
Province of residence (reference, Regina, Saskatchewan, and surrounding areas)	0.46 (0.28-0.77)	.003
Urbanicity (reference, rural)	3.03 (1.26-7.28)	.01
Referral to substance use disorder counseling (reference, no)	0.77 (0.48-1.24)	.29
Right-sided infection (reference, left-sided, bilateral, echo negative)	0.44 (0.27-0.71)	<.001
Congestive heart failure (reference, no)	2.32 (1.29-4.18)	.005

Abbreviation: aHR, adjusted hazard ratio.

^a^ aHRs were generated from a multivariable Cox proportional hazards regression model. The multivariable model was adjusted for age at time of admission, province of residence, urbanicity, referral to substance use disorder counseling, right-sided infection, and congestive heart failure.

### Mortality Among Men Who Inject Drugs

Among men, residing in London decreased the hazard of 5-year mortality (aHR, 0.54; 95% CI, 0.34-0.87; *P* = .01) compared with residing in Regina (Table 4). Having right-sided infection, compared with left-sided, bilateral, echo-negative infection, was protective among men only within 90 days of index admission (aHR, 0.22; 95% CI, 0.10-0.50; *P* < .001). Having CHF increased the hazard of 5-year mortality (aHR, 1.73; 95% CI, 1.07-2.79; *P* = .02).

**Table 4.** Multivariable Time-Dependent Cox Proportional for Factors Associated With 5-Year Mortality in Men Who Inject Drugs

Variable	aHR (95% CI)^a^	*P* value
Age at time of admission	1.01 (0.99-1.03)	.44
Province of residence (reference, Regina, Saskatchewan, and surrounding areas)	0.54 (0.34-0.87)	.01
Urbanicity (reference, rural)	0.77 (0.41-1.44)	.42
Referral to substance use disorder counseling (reference, no)	0.64 (0.39-1.07)	.09
Right-sided infection (reference, left-sided, bilateral, echo negative) by time		.005^b^
Right-sided infection <90 d	0.22 (0.10-0.50)	<.001
Right-sided infection 90-365 d	1.10 (0.44-2.74)	.83
Right-sided infection >365 d	1.06 (0.42-2.68)	.90
Congestive heart failure (reference, no)	1.73 (1.07-2.79)	.02

Abbreviation: aHR, adjusted hazard ratio.

^a^ aHRs were generated from a time-dependent multivariable Cox proportional hazards regression model. The multivariable model was adjusted for age at time of admission, province of residence, urbanicity, referral to substance use disorder counseling, interaction between right-sided infection and time, and congestive heart failure.

^b^ *P* value was derived from the log-likelihood ratio suggesting significant interaction term.

### Sensitivity Analysis

The imputed models demonstrated similar results, suggesting that bias was not introduced by excluding information provided by incomplete observations (eTable 6 in Supplement 2). Similar results were obtained for analyses of 1-year mortality (eTables 7-9 in Supplement 2) and the models including covariates involving surgery for all PWID and sex-stratified analyses (eTables 10-12 in Supplement 1).

### Pregnancy

Of 220 WWID, 11 (5.0%) were pregnant at admission, with 27.0% (3 of 11 women) dying (2 of 11 women during index hospitalization). Forty-six percent (5 of 11) of pregnancies were terminated; 60.0% (3 of 5 terminations) were elective termination and 40.0% (2 of 5 pregnancies) were miscarriages. Of 6 live births, the median (IQR) gestational age was 35.3 (28.1-38.6) weeks, with 83.0% (5 of 6 births) being vaginal births and 17.0% (1 of 6 births) being cesarean deliveries. Two of 6 newborns (33.0%) required neonatal intensive care unit care. Contraceptive use or nonuse was documented for only 12 of 220 women (5.5%).

## Discussion

To our knowledge, this is the largest cohort study of PWID with 2023 Duke-ISCVID criteria for definite IE, and our 5-year follow-up is the longest yet reported. Focusing on WWID with or at risk for IE is necessary for several reasons. First, although women made up 30.4% of all IE cases among non-PWIDs in our centers and comprise 32.7% of the PWID population,^8^ WWID comprised 51.2% of PWID with IE in our cohort. In a case-control study,^25^ we previously demonstrated an association between female sex and endocarditis among PWID. Differences in injecting practices from men (ie, women have a greater likelihood of having others perform the injecting and being among the last to inject, often with previously used equipment and/or more difficulty in accessing veins,^26^ and thus use of other sites) may lead to higher-risk practices but would require further study.^25,27,28^ Higher incidences of HIV and hepatitis C infection have been reported in WWID compared with men who inject drugs^29^ and are similarly attributed to differences in injecting practices, including greater equipment sharing. Whether specific physiological differences predispose WWID to IE or ascertainment bias leads to more common diagnoses (ie, greater health-seeking behavior or more-aggressive testing when presenting for care) will require further study. We noted that WWID with IE were younger than both non-PWID women and men with IE and men who inject drugs with IE. WWID were more likely to receive SUD counseling than men who inject drugs. Women were less likely to undergo surgery, corresponding with lower reported surgery rates among WWID in literature.^30^ This may be because of the large proportion of women in our cohort with right-sided infection, which is less likely to require surgery, and the reduced incidence of CHF and myocardial abscess compared with men.

Women were less likely than men to die within the first year in the univariable analysis but not at 5 years. Mortality between 1 and 5 years was numerically greater in women (but not significantly so). Lower short-term mortality among women may be related to increased frequency of right-sided heart disease, which is associated with lower short-term mortality and earlier presentation for care with a lower incidence of CHF and myocardial abscess at baseline. This phenomenon of late presentation of men for care has been noted in several other settings.^31,32,33^ Multivariable analysis correcting for side of infection and CHF did not demonstrate a difference in survival by sex; in rural areas, women had lower mortality than men, but the difference was not statistically significant. Multivariable analysis demonstrated a significant interaction between sex and urbanicity (*P* for interaction = .02), with lower mortality among women in rural areas than women in urban areas (eTable 7 in Supplement 1); however, no such pattern was seen for men. This outcome differed from data found in non-PWID with IE, for whom residing in urban areas is protective and believed to be due to increased access to health care.^34^ Urbanicity may be associated with homelessness, gender-related violence and abuse, risks associated with engagement in sex trade activities, and a lack of community support mechanisms.^35^ Moreover, community connection is especially important for Indigenous people,^36^ particularly for young Indigenous women.^37,38^ Such social structures are often unstable within urban settings, further complicating health outcomes for women.

SUD in pregnancy has more than quadrupled in the US between 1999 and 2014.^39^ In Canada, the proportion of infants born with neonatal abstinence syndrome increased 18% from 2013 to 2016,^40^ with approximately 0.51% of infants born with the condition from 2016 to 2017.^41^ IE in pregnancy prior to the opioid epidemic was rare, occurring in 1 of 100 000 pregnancies.^42^ We observed pregnancy in 5.0% of WWID with IE, with mortality occurring in 3 of 11 (27.0%) of the mothers (with 2 of 3 maternal deaths occurring during index hospitalization) and 25.0% (2 of 8) of not electively terminated fetuses. This is consistent with previous data suggesting that maternal mortality was not greater in maternal-associated IE than in non–maternal-associated IE, but that maternal and fetal outcomes are worse when IE complicates pregnancy compared with pregnancies not complicated by IE.^43^ Our data highlight the need for trauma-informed reproductive counseling and attention to contraceptive needs for WWID.^38,44,45^ Only 5.5% of these women had their use or nonuse of contraception documented; thus, we believe an important opportunity to provide access to contraception was missed. Lack of attention to the contraceptive needs of WWID has been previously documented.^46^

We compared the outcomes in London and Regina as they both have similar health care systems (with universal access to provincially funded medicine and composed of tertiary-care teaching hospitals based within moderately sized cities with populations of 250 000-500 000). However, these centers differ in that opiate substitution therapy (OST) was provided for inpatients in London, whereas only outpatient referral postdischarge was provided in Regina, and it was unclear whether patients attended this appointment. We demonstrated a significant interaction between the efficacy of SUD counseling and the location in which it was provided (*P* for interaction = .02) (Table 2). Among those referred for SUD counseling, there was lower mortality in London compared with Regina. Among those not referred, there was no significant difference by location. A previous study^47^ showed improvement in mortality in PWID with IE while receiving OST, but only while receiving therapy. We suspect that provision of inpatient OST and SUD counseling in London may have led to better outcomes compared with referral to outpatient SUD services postdischarge with no inpatient OST as practiced in Regina. Many patients referred to outpatient services fail to attend, and we suspect providing SUD care while still an inpatient may lead to better long-term retention in care. We suspect this marked difference in how SUD referrals are provided in the 2 centers is the reason why we did not see a benefit in overall mortality with SUD referral at 5 years. There was no significant interaction between the efficacy of SUD referral and sex (eTable 7 in Supplement 1). Regina has recently implemented wrap-around support that includes SUD counseling and OST for inpatients. Further investigation is needed to determine whether this will improve outcomes.

A strength of our study was using citywide medical records, allowing for exceptional long-term follow-up of our cohort with 96.5% having 1-year and 80.7% having 5-year survival data. We found a very high 48.7% 5-year mortality in this PWID with IE population. This was much higher than that seen in the few long-term outcome studies of non–PWID-associated IE,^48,49,50^ despite the fact non-PWID with IE tend to be more than 20 years older than PWID and have underlying cardiac disease.^2^ Most deaths beyond 1 year were likely related to social and medical complications of SUD, including complications of reinfection. This highlights the extreme vulnerability of this population and the need for improved long-term addiction therapies.

### Limitations

This study has limitations that should be mentioned. There is a lack of information on both ethnicity and gender, with most medical records reporting only male or female sex. It is estimated that the proportion of people who have injected drugs in their lifetime in the US is 2.6% (3.6% for men and 1.6% for women).^10^ Similarly, data from a recent national survey in Canada estimated that the PWID population comprises 65.0% cisgender men, 32.7% cisgender women, 1.0% transgender women, and 0.7% transgender men.^8^ This suggests that, although further study regarding nonbinary persons with IE is important, the data are unlikely to change the overall conclusions of our analysis.

Other limitations include limited ethnographic data. Indigenous status data are not routinely collected at either site, and individuals from other minoritized racial and ethnic groups are not common at either center. Indigenous persons make up a higher percentage of the population in Saskatchewan than Ontario.^51^ It is crucial to acknowledge the major impact that colonization and colonial policies on matriarchal societies have had on social and health disparities experienced by Indigenous peoples, especially women, in Canada and elsewhere.^38^ Adhering to the recommendations of the Truth and Reconciliation Commission of Canada^52^ is important to address the social and health disparities of Indigenous peoples across Canada. Cause-specific mortality was not available for many patients because a large number died outside of the hospital, with only obituary data available. We focused on long-term outcomes and, by necessity, enrolled patients before the COVID-19 pandemic. There was an increase in overall drug use during the pandemic and a disproportionate impact on minoritized racial and ethnic groups.^53,54^ Further studies of recent endocarditis epidemiology are needed. Our data are based on a Canadian context where there is universal access to health care and where most patients with IE are treated as inpatients. Furthermore, most of the cases were from London. Therefore, generalization to other centers may be limited. This study was based on a retrospective record review containing routinely collected data. There remains the risk of an ecological fallacy with using area codes to attribute population-level characteristics regarding urbanicity to individuals, and residual confounding, including confounding by indication or immortal time bias for patients referred to SUD counseling (although we sought to minimize this by including data from only the index admission).

## Conclusions

Although women comprise one-third of PWID, they comprise one-half of those with IE in our centers. The reason for the disproportionately high incidence of IE among WWID needs further study. Women were younger and more likely to develop right-sided IE than men. Higher long-term mortality was noted in WWID with IE living in urban centers, suggesting the need for greater social support and harm reduction services. Fetal outcomes were poor in pregnant WWID with IE. Contraception counseling needs to be prioritized in this population with high pregnancy rates. Among all patients referred for SUD services, being in a center where these were provided as an inpatient was associated with lower long-term mortality.

## Notes

Supplement 1. **eMethods. **Description of Study Measures **eTable 1.** Cross Tabulation of Persons Who Inject Drugs From Index Hospitalization Stratified by Patient Sex **eTable 2.** Additional Baseline Characteristics of Persons Who Inject Drugs From Index Hospitalization Stratified by Patient Sex **eTable 3.** Cross Tabulation of Recurrent Infective Endocarditis in Persons Who Inject Drugs by Patient Sex **eTable 4.** Death Characteristics Among People Who Inject Drugs With Infective Endocarditis Stratified by Patient Sex **eTable 5.** Baseline Characteristics of People Who Inject Drugs From Index Hospitalization Stratified by 1-Year and 5-Year Follow-up **eTable 6.** Substantive Model Compatible Fully Conditional Specification Imputation of Baseline Covariate Effects of the Multivariable Cox Proportional for Factors Associated With Five-Year Mortality Among Persons Who Inject Drugs **eTable 7.** Multivariable Time-Dependent Cox Proportional for Factors Associated With One-Year Mortality in People Who Inject Drugs **eTable 8.** Multivariable Cox Proportional for Factors Associated With One-Year Mortality in Women Who Inject Drugs **eTable 9.** Multivariable Time-Dependent Cox Proportional for Factors Associated With One-Year Mortality in Men Who Inject Drugs **eTable 10.** Sensitivity Analysis: Multivariable Time-Dependent Cox Proportional for Factors Associated With Five-Year Mortality in People Who Inject Drugs Including Surgery as a Covariate **eTable 11.** Sensitivity Analysis: Multivariable Time-Dependent Cox Proportional for Factors Associated With Five-Year Mortality in Women Who Inject Drugs Including Surgery as a Covariate **eTable 12.** Sensitivity Analysis: Multivariable Time-Dependent Cox Proportional for Factors Associated With Five-Year Mortality in Men Who Inject Drugs Including Surgery as a Covariate **eFigure.** Causal Diagram
eReferences

Supplement 2.
Data Sharing Statement
