# Supplementary material for: Infective Endocarditis Among Women Who Inject Drugs
Source: JAMA Netw Open. 2024 Oct 4;7(10):e2437861. doi: 10.1001/jamanetworkopen.2024.37861 (PMC11452813; doi:10.1001/jamanetworkopen.2024.37861)
Supplement: Supplement 1. — eMethods. Description of Study Measures eTable 1. Cross Tabulation of Persons Who Inject Drugs From Index Hospitalization Stratified by Patient Sex eTable 2. Additional Baseline Characteristics of Persons Who Inject Drugs From Index Hospitalization Stratified by Patient Sex eTable 3. Cross Tabulation of Recurrent Infective Endocarditis in Persons Who Inject Drugs by Patient Sex eTable 4. Death Characteristics Among People Who Inject Drugs With Infective Endocarditis Stratified by Patient Sex eTable 5. Baseline Characteristics of People Who Inject Drugs From Index Hospitalization Stratified by 1-Year and 5-Year Follow-up eTable 6. Substantive Model Compatible Fully Conditional Specification Imputation of Baseline Covariate Effects of the Multivariable Cox Proportional for Factors Associated With Five-Year Mortality Among Persons Who Inject Drugs eTable 7. Multivariable Time-Dependent Cox Proportional for Factors Associated With One-Year Mortality in People Who Inject Drugs eTable 8. Multivariable Cox Proportional for Factors Associated With One-Year Mortality in Women Who Inject Drugs eTable 9. Multivariable Time-Dependent Cox Proportional for Factors Associated With One-Year Mortality in Men Who Inject Drugs eTable 10. Sensitivity Analysis: Multivariable Time-Dependent Cox Proportional for Factors Associated With Five-Year Mortality in People Who Inject Drugs Including Surgery as a Covariate eTable 11. Sensitivity Analysis: Multivariable Time-Dependent Cox Proportional for Factors Associated With Five-Year Mortality in Women Who Inject Drugs Including Surgery as a Covariate eTable 12. Sensitivity Analysis: Multivariable Time-Dependent Cox Proportional for Factors Associated With Five-Year Mortality in Men Who Inject Drugs Including Surgery as a Covariate eFigure. Causal Diagram eReferences [file jamanetwopen-e2437861-s001.pdf]

Supplemental Online Content

Adams JA, Spence C, Shojaei E, et al. Infective endocarditis in women who inject drugs. *JAMA Netw Open*. 2024;7(10):e2437861. doi:10.1001/jamanetworkopen.2024.37861

eMethods. Description of Study Measures

eTable 1. Cross Tabulation of Persons Who Inject Drugs From Index Hospitalization Stratified by Patient Sex

eTable 2. Additional Baseline Characteristics of Persons Who Inject Drugs From Index Hospitalization Stratified by Patient Sex

eTable 3. Cross Tabulation of Recurrent Infective Endocarditis in Persons Who Inject Drugs by Patient Sex

eTable 4. Death Characteristics Among People Who Inject Drugs With Infective Endocarditis Stratified by Patient Sex

eTable 5. Baseline Characteristics of People Who Inject Drugs From Index Hospitalization Stratified by 1-Year and 5-Year Follow-up

eTable 6. Substantive Model Compatible Fully Conditional Specification Imputation of Baseline Covariate Effects of the Multivariable Cox Proportional for Factors Associated With Five-Year Mortality Among Persons Who Inject Drugs

eTable 7. Multivariable Time-Dependent Cox Proportional for Factors Associated With One-Year Mortality in People Who Inject Drugs

eTable 8. Multivariable Cox Proportional for Factors Associated With One-Year Mortality in Women Who Inject Drugs

eTable 9. Multivariable Time-Dependent Cox Proportional for Factors Associated With One-Year Mortality in Men Who Inject Drugs

eTable 10. Sensitivity Analysis: Multivariable Time-Dependent Cox Proportional for Factors Associated With Five-Year Mortality in People Who Inject Drugs Including Surgery as a Covariate

eTable 11. Sensitivity Analysis: Multivariable Time-Dependent Cox Proportional for Factors Associated With Five-Year Mortality in Women Who Inject Drugs Including Surgery as a Covariate

eTable 12. Sensitivity Analysis: Multivariable Time-Dependent Cox Proportional for Factors Associated With Five-Year Mortality in Men Who Inject Drugs Including Surgery as a Covariate

eFigure. Causal Diagram

eReferences

This supplemental material has been provided by the authors to give readers additional information about their work.

eMethods. Description of Study Measures

Demographic variables included patient age, sex, province of residence, Indigenous status, and urbanicity. Indigenous status was recorded using the "legal standing of a person who is registered under the Indian Act"[1] which is logged for billing purposes. Urbanicity refers to the patient's location within a metropolitan area, derived using the first three characters of a patient's postal code. The second character of a Canadian postal code is indicative of whether a patient lives in an urban or rural setting. Postal codes with a second character of "0" are defined as rural settings.[2] Discharge diagnosis of IE was determined by ICD-10 codes (I33.0 Acute and Subacute infective endocarditis, I33.9 Acute and subacute endocarditis, unspecified, I38 Endocarditis, valve unspecified and I39.8 Endocarditis, valve unspecified, in diseases classified elsewhere).

Drug misuse was defined as self-reported injection drug use within three months of admission. The type of substance misused was treated as a categorical variable consisting of mono-substance misuse of opiates, mono-substance misuse of stimulants, polysubstance misuse, or other (unknown injected substance or antidepressant). In settings where urine toxicology tests demonstrated substances not reported by the patient, the substances detected in the urine test were included as drugs used. IE infection location consisted of the primary valve and heart region infected. IE infection was defined as a left-sided, right-sided, or bilateral infection, or negative echocardiography (echo-negative) IE. Left-sided IE was defined as an infection affecting left-sided cardiac structures, whereas right-sided IE was defined as an infection affecting right-sided cardiac structures. IE affecting both left- and right-sided structures were defined as a bilateral infection. Echo-negative infection IE was defined as definite infection as defined by the 2023 Duke-ISCVID criteria but vegetations were not seen on echocardiography.[3] Causative organisms were defined as microbial agents responsible for causing the development of IE. IE cases where more than one primary causative organism was found were defined as polymicrobial. Culture negative infection was defined as fulfilling the 2023 Duke-ISCVID criteria for definite IE with blood cultures showing the absence of causative organisms.[3] Surgery was defined as undergoing valve repair or replacement, debridement of a myocardial abscess or removal of an intracardiac device. Heart failure was diagnosed by the attending team. Cardiac complications consisted of myocardial abscess, aortic root abscess, congestive heart failure, and conduction delay. Vascular complications included ischemic stroke, intracerebral hemorrhage, mycotic aneurysm, and septic pulmonary emboli. Cause of death was categorized as either sepsis, cardiac-related, respiratory-related, embolism, stroke, overdose or other. Antibiotic administration route was defined as either intravenous/intramuscular only (≥28 days of parenteral therapy) or a combination of intravenous and oral (<28 days of intravenous therapy with oral therapy for the remainder [>14 days]). Recurrent infections were defined as infections occurring greater than six months apart or caused by a different causative organism and with new cardiac vegetation.[4–6] Infections that do not fulfill the recurrent disease criteria were defined as relapsed IE.

© 2024 Adams JA et al. *JAMA Network Open.*

eTable 1. Cross Tabulation of Persons Who Inject Drugs From Index Hospitalization Stratified by Patient Sex

	Total (n=762)	Women (n=321)	Men (n=441)	P
Person who injects drugs, No. (%)				
No	332 (43.6)	101 (31.5)	231 (52.4)	<.001
Yes	430 (56.4)	220 (68.5)	210 (47.6)	

Abbreviations: No., number

© 2024 Adams JA et al. *JAMA Network Open.*

eTable 2. Additional Baseline Characteristics of Persons Who Inject Drugs From Index Hospitalization[a] Stratified by Patient Sex

	Total (n=430)	Women (n=220)	Men (n=210)
HIV status, No. (%)			
No	309 (86.8)	168 (88.9)	141 (84.4)
Yes	47 (13.2)	21 (11.1)	26 (15.6)
Unknown	74	31	43
Hepatitis C status, No. (%)			
Negative	83 (21.2)	45 (22.3)	38 (20.1)
Positive	308 (78.8)	157 (77.7)	151 (79.9)
Unknown	39	18	21
Category of injection substances used, No. (%)[b]			
Opiate only	122 (31.0)	59 (28.0)	60 (34.4)
Stimulant only	45 (11.4)	25 (11.9)	20 (10.9)
Polysubstance (opiates and stimulants)	226 (57.4)	126 (59.7)	100 (54.6)
Benzodiazepine only	1 (0.3)	1 (0.5)	0 (0.0)
Unknown	36	9	27
Length of Hospital Stay, median (IQR), days	27.0 (12.0–46.0)	29.0 (13.0–47.5)	23.5 (12.0–45.0)
Peripherally inserted central catheter misuse, No. (%)			
No	242 (60.1)	115 (55.3)	127 (65.1)
Yes	109 (27.1)	65 (31.3)	44 (22.6)
No peripherally inserted central catheter in place	52 (12.9)	28 (13.5)	24 (12.3)
Unknown	27	12	15
Route of antibiotic administration, No. (%)			
Intravenous or intramuscular	346 (80.5)	173 (78.6)	173 (82.4)
Intravenous and oral	84 (19.5)	47 (21.4)	37 (17.6)
Left against medical advice, No. (%)			
No	348 (80.9)	182 (82.7)	166 (79.1)
Yes	82 (19.1)	38 (17.3)	44 (21.0)
Infectious disease consultation, No. (%)			
No	19 (4.8)	8 (3.9)	11 (5.8)
Yes	378 (95.2)	198 (96.1)	180 (94.2)
Unknown	33	14	19
Causative organism, No. (%)			
Viridans group streptococci	18 (4.2)	5 (2.3)	13 (6.3)
Enterococcus species	18 (4.2)	6 (2.8)	12 (5.8)
Gram-negative bacilli	13 (3.1)	5 (2.3)	8 (3.9)
Fungal	3 (0.7)	2 (0.92)	1 (0.48)
Non-viridans group streptococci	15 (3.5)	7 (3.2)	8 (3.9)
Methicillin-sensitive *Staphylococcus aureus*	227 (53.4)	122 (55.7)	105 (50.7)
Methicillin-resistant *Staphylococcus aureus*	100 (23.5)	56 (25.7)	44 (21.3)
Other	4 (0.9)	3 (1.4)	1 (0.5)
Polymicrobial	24 (5.6)	10 (4.6)	14 (6.8)
Culture negative	3 (0.7)	2 (0.9)	1 (0.5)
Unknown	5	2	3

Abbreviations: No, number
[a] During first episode
[b]Mono substance misuse of either opiates, stimulants or benzodiazepines

© 2024 Adams JA et al. *JAMA Network Open.*

eTable 3. Cross Tabulation of Recurrent Infective Endocarditis in Persons Who Inject Drugs by Patient Sex

	Total (n=430)	Women (n=220)	Men (n=210)	P value
Recurrent infections, No. (%)				.26
No	335 (78.1)	167 (49.9)	168 (50.2)	
Yes	94 (21.9)	53 (56.4)	41 (43.6)	
Unknown	1	0	1	
Number of recurrent episodes				
0 (single episode only)	335 (78.1)	167 (49.9)	168 (50.2)	
1	58 (13.5)	33 (56.9)	25 (43.1)	
2	23 (5.4)	12 (52.2)	11 (47.8)	
3	11 (2.6)	7 (63.6)	4 (36.4)	
4	2 (0.5)	1 (50.0)	1 (50.0)	
Unknown	1	0	1	

Abbreviation: No., number

eTable 4. Death Characteristics Among People Who Inject Drugs With Infective Endocarditis Stratified by Patient Sex

	Total (n=430)	Women (n=220)	Men (n=210)	P[a]
Died within 1 year following admission, No. (%)				.03
No	299 (72.1)	162 (54.2)	137 (45.8)	
Yes	116 (28.0)	49 (42.2)	67 (57.8)	
Total	415 (100.0)	211 (100.0)	204 (1.00)	
Lost to follow-up	15	9	6	
	**Total (n=116)**	**Women (n=49)**	**Men (n=67)**	
1-year endocarditis-related mortality, No. (%)[b]				
No	10 (9.4)	4 (40.0)	6 (60.0)	
Yes	96 (90.6)	42 (43.8)	54 (56.3)	
Total	106 (100.0)	46 (100.0)	60 (100.0)	
Unknown	10	3	7	
1-year mortality underlying cause of death, No. (%)[c]				
Heart failure	15 (15.5)	8 (53.3)	7 (46.7)	
Medical assistance in dying	1 (1.0)	1 (100.0)	0 (0.0)	
Respiratory failure	1 (1.0)	1 (100.0)	0 (0.0)	
Sepsis	63 (65.0)	28 (44.4)	35 (55.6)	
Stroke	16 (16.5)	5 (31.3)	11 (20.4)	
Other	1 (1.0)	0 (0.0)	1 (100.0)	
Total	97 (100.0)	43 (100.0)	54 (100.0)	
Unknown	19	6	13	
	**Total (n=430)**	**Women (n=220)**	**Men (n=210)**	**P**
Died within 1–5 years following admission, No. (%)				.51

© 2024 Adams JA et al. *JAMA Network Open.*

No	178 (77.1)	95 (53.4)	83 (46.6)	
Yes	53 (22.9)	31 (58.5)	22 (41.5)	
Total	231 (100.0)	126 (100.0)	105 (100.0)	
Lost to follow-up	68	36	32	
	**Total (n=53)**	**Women (n=31)**	**Men (n=22)**	
1–5 years endocarditis-related mortality, No. (%)[b]				
No	10 (28.6)	7 (70.0)	3 (30.0)	
Yes	25 (71.4)	13 (52.0)	12 (48.0)	
Total	35 (100.0)	20 (100.0)	15 (100.0)	
Unknown	18	11	7	
Underlying cause of death, No. (%)[c,d]				
Heart failure	4 (16.7)	3 (75.0)	1 (25.0)	
Respiratory failure	2 (8.3)	1 (50.0)	1 (50.0)	
Sepsis	16 (66.7)	8 (50.0)	8 (50.0)	
Stroke	2 (8.3)	1 (50.0)	1 (50.0)	
Total	24 (100.0)	13 (100.0)	11 (100.0)	
Unknown	29	18	11	
	**Total (n=430)**	**Women (n=220)**	**Men (n=210)**	**P**
Died within 5 years following admission, No. (%)				.26
No	178 (51.3)	95 (53.4)	83 (46.6)	
Yes	169 (48.7)	80 (47.3)	89 (52.7)	
Total	347 (100.0)	175 (100.0)	172 (100.0)	
Lost to follow-up	83	45	38	

Abbreviation: No., number

[a]Pearson's chi-square test

[b]Mortality directly caused by complications of IE such as sepsis, heart failure, arrhythmia, intracerebral and systemic embolization (non-stroke).

[c]Includes patient deaths during and after present episode of infective endocarditis.

[d]Deaths occurring beyond 1 year were identified by cause but are not likely related to the index episode of endocarditis

© 2024 Adams JA et al. *JAMA Network Open.*

eTable 5. Baseline Characteristics of People Who Inject Drugs From Index Hospitalization[a] Stratified by 1-Year and 5-Year Follow-up

		1-Year Follow-up			5-Year Follow-up		
**Characteristic**	**Total (n=430)**	**Yes (n=415)**	**No (n=15)**	**P**	**Yes (n=347)**	**No (n=83)**	**P**
Age, median [IQR]	**35**.0 [28.0–43.0]	35.0 [28.0–43.0]	36.0 [33.0–43.0]	.28[b]	36.0 [29.0–43.0]	34.0 [28.0–43.0]	.28[b]
Sex, No. (%)				.60[c]			.54[d]
Women	220 (51.2)	221 (95.9)	9 (4.1)		175 (79.6)	45 (20.5)	
Men	210 (48.8)	204 (97.1)	6 (2.9)		172 (51.9)	38 (18.1)	
Province of residence, No. (%)				.57[c]			<.001[d]
Saskatchewan (Regina)	120 (27.9)	115 (95.8)	5 (4.2)		78 (65.0)	42 (35.0)	
Ontario (London)	310 (72.1)	300 (96.8)	10 (3.2)		269 (86.8)	41 (13.2)	
Urbanicity, No. (%)				.43[c]			.29[d]
Rural	57 (13.3)	54 (94.7)	3 (5.3)		43 (75.4)	14 (24.6)	
Urban	371 (86.7)	359 (96.8)	12 (3.2)		302 (81.4)	69 (18.6)	
Unknown	2	2	0		2	0	
Site of infection, No. (%)				.28[c]			.09[d]
Left-sided/bilateral/echo-negative	159 (37.0)	156 (98.1)	3 (1.9)		135 (84.9)	24 (15.1)	
Right-sided	271 (63.0)	259 (95.6)	12 (4.4)		212 (78.2)	59 (21.8)	
Referral to substance use disorder counseling, No. (%)				.42[c]			.19[d]
No	258 (60.6)	247 (95.7)	11 (4.3)		213 (82.6)	45 (17.4)	
Yes	168 (39.4)	164 (97.6)	4 (2.4)		45 (17.4)	38 (22.6)	
Unknown	4	4	0		4	0	
Congestive heart failure, No. (%)				.14[c]			.04[d]
No	334 (82.3)	321 (96.1)	13 (3.9)		262 (78.4)	72 (21.6)	
Yes	72 (17.7)	72 (100.0)	0 (0.0)		64 (88.9)	8 (11.1)	
Unknown	24	22	2		21	3	
Surgery, No. (%)				.71[c]			.56[d]
No	364 (84.7)	352 (96.7)	12 (3.3)		292 (80.2)	72 (19.8)	
Yes	66 (15.4)	63 (95.5)	3 (4.6)		55 (15.4)	11 (16.7)	

Abbreviation: No., number
[a] During first episode
[b] Wilcoxon two-sample test
[c] Fisher's exact test
[d] Pearson's chi-square test

© 2024 Adams JA et al. *JAMA Network Open.*

eTable 6. Substantive Model Compatible Fully Conditional Specification Imputation of Baseline Covariate Effects of the Multivariable Cox Proportional for Factors Associated With Five-Year Mortality Among Persons Who Inject Drugs

	Complete Case Analysis		Substantive Model Compatible Fully Conditional Specification Imputation	
	aHR[a]	95% CI	aHR[a]	95% CI
Age at time of admission	1.01	1–1.03	1.01	0.99–1.03
Province of residence (ref=Regina, SK and surrounding areas)	0.66	0.42–1.03	0.70	0.45–1.08
Urbanicity (ref=rural)	2.86	1.22–6.74[b]	2.48	1.17–5.25[b]
Referral to substance use disorder counseling (ref=no)	1.19	0.64–2.19	1.30	0.71–2.38
Sex (ref=female)	3.07	1.08–8.74 [b]	2.70	1.04–7.00[b]
Right-sided infection (ref=left-sided/bilateral/echo negative)	0.47	0.33–0.67 [b]	0.45	0.33–0.63[b]
Congestive heart failure (ref=no)	1.87	1.29–2.7 [b]	1.80	1.24–2.6[b]
Sex × Urbanicity	0.29	0.1–0.83 [b]	0.35	0.13–0.9[b]
Sex × Referral to substance use disorder counseling	0.90	0.45–1.8	0.81	0.41–1.60
Province of residence × Referral to substance use disorder counseling	0.47	0.23–0.96 [b]	0.43	0.22–0.86[b]

Abbreviations: HR, hazard ratio; CI, confidence interval; SK, Saskatchewan

[a]Adjusted HRs were generated from s multivariable Cox proportional hazards regression model. The multivariable model was adjusted for age at time of admission, province of residence, urbanicity, referral to substance use disorder counseling, sex, right-sided infection, congestive heart failure, interaction between sex and urbanicity, interaction between sex and referral to substance use disorder counseling, and interaction between province of residence and referral to substance use disorder counseling.

[b]95% CIs that do not include '1.00' suggest statistical significance

© 2024 Adams JA et al. *JAMA Network Open.*

eTable 7. Multivariable Time-Dependent Cox Proportional for Factors Associated With One-Year Mortality in People Who Inject Drugs

	aHR[a]	95% CI		P
Age at time of admission	1.02	1.00	1.04	.13
Province of residence (ref=Regina, SK and surrounding areas)	0.50	0.33	0.75	<.001
Sex × Urbanicity				.03[b]
Females vs. males in urban areas	1.08	0.68	1.69	.74
Females vs. males in rural areas	0.27	0.07	0.98	.05
Residing in urban areas vs. rural areas among women	2.97	0.91	9.73	.07
Residing in urban areas vs. rural areas among men	0.73	0.37	1.45	.37
Referral to substance use disorder counseling (ref=no)	0.60	0.39	0.93	.02
Right-sided infection (ref=left-sided/bilateral/echo negative) × time				.004[b]
Right-sided infection <90 days	0.24	0.14	0.43	<.001
Right-sided infection 90–180 days	0.89	0.32	2.44	.81
Right-sided infection >180 days	1.12	0.42	2.97	.82
Congestive heart failure (ref=no)	1.85	1.20	2.85	.005

Abbreviations: HR, hazard ratio; CI, confidence interval; SK, Saskatchewan

[a]Adjusted HRs were generated from a time-dependent multivariable Cox proportional hazards regression model. The multivariable model was adjusted for age at time of admission, province of residence, interaction between urbanicity and sex, referral to substance use disorder counseling, interaction between right-sided infection and time, and congestive heart failure.

[b] P-values derived from the log-likelihood ratio suggesting significant interaction term.

© 2024 Adams JA et al. *JAMA Network Open.*

eTable 8. Multivariable Cox Proportional for Factors Associated With One-Year Mortality in Women Who Inject Drugs

	aHR[a]	95% CI		P
Age at time of admission	1.03	0.99	1.07	.15
Province of residence (ref=Regina, SK and surrounding areas)	0.50	0.26	0.95	.03
Urbanicity (ref=rural)	3.72	1.08	12.73	.04
Referral to substance use disorder counseling (ref=no)	0.71	0.38	1.34	.30
Right-sided infection (ref=left-sided/bilateral/echo negative)	0.38	0.20	0.73	.003
Congestive heart failure (ref=no)	2.96	1.48	5.90	.002

Abbreviations: HR, hazard ratio; CI, confidence interval; SK, Saskatchewan

[a]Adjusted HRs were generated from a multivariable Cox proportional hazards regression model. The multivariable model was adjusted for age at time of admission, province of residence, urbanicity, referral to substance use disorder counseling, right-sided infection, and congestive heart failure.

© 2024 Adams JA et al. *JAMA Network Open.*

eTable 9. Multivariable Time-Dependent Cox Proportional for Factors Associated With One-Year Mortality in Men Who Inject Drugs

	aHR[a]	95% CI		P
Age at time of admission	1.01	0.99	1.04	.30
Province of residence (ref=Regina, SK and surrounding areas)	0.52	0.30	0.88	.01
Urbanicity (ref=rural)	0.71	0.35	1.41	.32
Referral to substance use disorder counseling (ref=no)	0.47	0.25	0.90	.02
Right-sided infection (ref=left-sided/bilateral/echo negative) × time				.009[b]
Right-sided infection <90 days	0.24	0.11	0.53	<.001
Right-sided infection 90–180 days	0.58	0.15	2.21	.43
Right-sided infection >180 days	2.12	0.56	8.06	.27
Congestive heart failure (ref=no)	1.46	0.85	2.53	.17

Abbreviations: HR, hazard ratio; CI, confidence interval; SK, Saskatchewan
[a]Adjusted HRs were generated from a time-dependent multivariable Cox proportional hazards regression model. The multivariable model was adjusted for age at time of admission, province of residence, urbanicity, referral to substance use disorder counseling, interaction between right-sided infection and time, and congestive heart failure.
[b] P-values derived from the log-likelihood ratio suggesting significant interaction term.

© 2024 Adams JA et al. *JAMA Network Open.*

.eTable 10. Sensitivity Analysis: Multivariable Time-Dependent Cox Proportional for Factors Associated With Five-Year Mortality in People Who Inject Drugs Including Surgery as a Covariate

	aHR[a]	95% CI		P
Age at time of admission	1.01	1.00	1.03	.11
Province of residence (ref=Regina, SK and surrounding areas) × referral to substance use disorder counseling				.01[b]
Residing in London, ON and surrounding areas vs. Regina, SK and surrounding areas among those who were referred to substance use disorder counseling	0.29	0.16	0.50	<.001
Residing in London, ON and surrounding areas vs. Regina, SK and surrounding areas among those who were not referred to substance use disorder counseling	0.71	0.45	1.11	.13
Sex × Urbanicity				.03[b]
Females vs. males in urban areas	1.17	1.71	0.81	.40
Females vs. males in rural areas	0.39	1.07	0.15	.07
Residing in urban regions vs. rural regions in women	2.75	1.17	6.44	.02
Residing in urban regions vs. rural regions in men	0.92	0.49	1.72	.80
Right-sided infection (ref=left-sided/bilateral/echo negative) × time				.004[b]
Right-sided infection <90 days	0.23	0.13	0.41	<.001
Right-sided infection 90–365 days	0.74	0.36	1.51	.40
Right-sided infection >365 days	0.73	0.39	1.38	.33
Congestive heart failure (ref=no)	1.73	1.19	2.51	.004
Surgery (ref=no)	1.05	0.57	1.90	.89

Abbreviations: HR, hazard ratio; CI, confidence interval; SK, Saskatchewan; ON, Ontario

[a]Adjusted HRs were generated from a time-dependent multivariable Cox proportional hazards regression model. The multivariable model was adjusted for age at time of admission, interaction between referral to substance us disorder counseling and time, interaction between right-sided infection and surgery, interaction between right-sided infection and time, interaction between urbanicity and time, interaction between province of residence and referral to substance use disorder counseling.

[b] P-values derived from the log-likelihood ratio suggesting significant interaction term.

© 2024 Adams J et al. *JAMA Network Open.*

eTable 11. Sensitivity Analysis: Multivariable Time-Dependent Cox Proportional for Factors Associated With Five-Year Mortality in Women Who Inject Drugs Including Surgery as a Covariate

	aHR[a]	95% CI		P
Age at time of admission	1.02	0.99	1.05	.12
Province of residence (ref=Regina, SK and surrounding areas)	0.46	0.28	0.76	.002
Urbanicity (ref=rural)	3.03	1.27	7.25	.01
Referral to substance use disorder counseling (ref=no)	0.77	0.47	1.24	.28
Right-sided infection (ref=left-sided/bilateral/echo negative)	0.45	0.27	0.76	.003
Congestive heart failure (ref=no)	2.34	1.30	4.23	.005
Surgery (ref=no)	1.16	0.51	2.64	.72

Abbreviations: HR, hazard ratio; CI, confidence interval; SK, Saskatchewan
[a]Adjusted HRs were generated from a multivariable Cox proportional hazards regression model. The multivariable model was adjusted for age at time of admission, province of residence, urbanicity, referral to substance use disorder counseling, right-sided infection, congestive heart failure and surgery.

© 2024 Adams J et al. *JAMA Network Open.*

eTable 12. Sensitivity Analysis: Multivariable Time-Dependent Cox Proportional for Factors Associated With Five-Year Mortality in Men Who Inject Drugs Including Surgery as a Covariate

	aHR[a]	95% CI		P
Age at time of admission	1.01	0.99	1.03	.37
Province of residence (ref=Regina, SK and surrounding areas)	0.54	0.34	0.86	.01
Urbanicity (ref=rural)	0.80	0.42	1.50	.48
Referral to substance use disorder counseling (ref=no)	0.66	0.39	1.09	.10
Right-sided infection (ref=left-sided/bilateral/echo negative) × time				.03[b]
Right-sided infection <90 days	0.23	0.10	0.51	<.001
Right-sided infection 90–365 days	1.16	0.46	2.90	.76
Right-sided infection >365 days	1.13	0.44	2.91	.80
Congestive heart failure (ref=no)	1.69	1.05	2.74	.03
Surgery (ref=no)	1.27	0.66	2.43	.48

Abbreviations: HR, hazard ratio; CI, confidence interval; SK, Saskatchewan

[a]Adjusted HRs were generated from a time-dependent multivariable Cox proportional hazards regression model. The multivariable model was adjusted for age at time of admission, province of residence, urbanicity, referral to substance use disorder counseling, interaction between right-sided infection and time, congestive heart failure and surgery.

© 2024 Adams J et al. *JAMA Network Open.*

eFigure. Causal Diagram

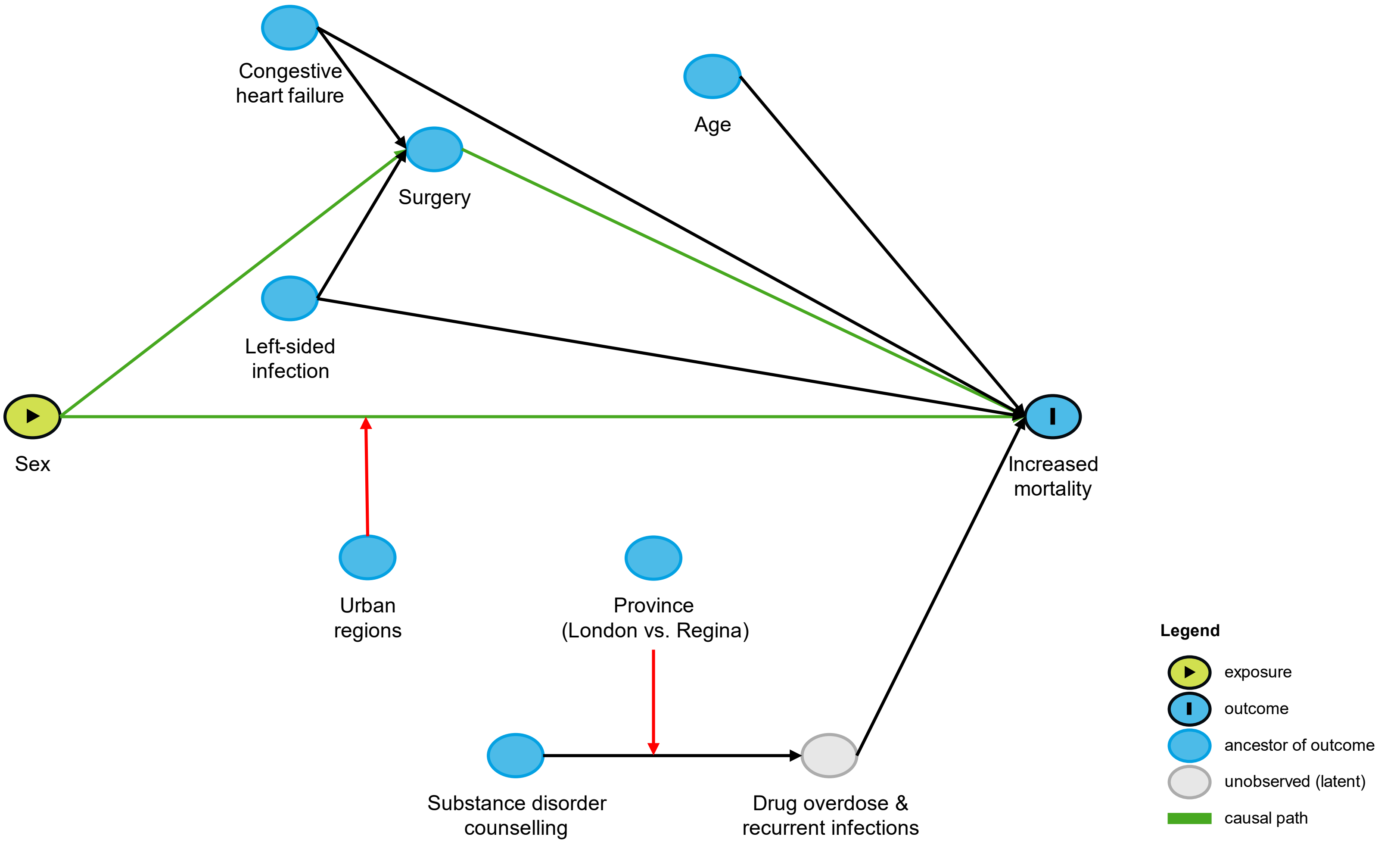

© 2024 Adams J et al. *JAMA Network Open.*

eReferences

1. Government of Canada. About Indian status. Government of Canada. November 3, 2008. Accessed March 16, 2023. https://www.sac-isc.gc.ca/eng/1100100032463/1572459644986

2. Statistics Canada. Postal Code Conversion File (PCCF), Reference Guide. Section 4 Technical specifications. Statistics Canada. July 20, 2011. Accessed March 16, 2023. https://www150.statcan.gc.ca/n1/pub/92-153-g/2011002/tech-eng.htm

3. Fowler VG, Durack DT, Selton-Suty C, et al. The 2023 Duke-International Society for Cardiovascular Infectious Diseases Criteria for Infective Endocarditis: Updating the Modified Duke Criteria. *Clin Infect Dis Off Publ Infect Dis Soc Am*. 2023;77(4):518-526. doi:10.1093/cid/ciad271

4. Rodger L, Shah M, Shojaei E, Hosseini S, Koivu S, Silverman M. Recurrent Endocarditis in Persons Who Inject Drugs. *Open Forum Infect Dis*. 2019;6(10):ofz396. doi:10.1093/ofid/ofz396

5. Baddour LM. Twelve-year review of recurrent native-valve infective endocarditis: a disease of the modern antibiotic era. *Rev Infect Dis*. 1988;10(6):1163-1170. doi:10.1093/clinids/10.6.1163

6. Lossos IS, Oren R. Recurrent infective endocarditis. *Postgrad Med J*. 1993;69(816):816-818.

© 2024 Adams J et al. *JAMA Network Open.*
