# Supplementary material for: Infective Endocarditis Among Women Who Inject Drugs
Source: JAMA Netw Open. 2024 Oct 4;7(10):e2437861. doi: 10.1001/jamanetworkopen.2024.37861 (PMC11452813; doi:10.1001/jamanetworkopen.2024.37861)
Supplement: Supplement 2. — Data Sharing Statement [file jamanetwopen-e2437861-s002.pdf]

Data Sharing Statement

Adams. Infective Endocarditis In Women Who Inject Drugs. *JAMA Netw Open*. Published October 04, 2024. doi:10.1001/jamanetworkopen.2024.37861

Data
Data available: No
